# Exploring differential item functioning in the SF-36 by demographic, clinical, psychological and social factors in an osteoarthritis population

**DOI:** 10.1186/1471-2474-14-346

**Published:** 2013-12-11

**Authors:** Beth Pollard, Marie Johnston, Diane Dixon

**Affiliations:** 1 Aberdeen Health Psychology Group, University of Aberdeen, 2nd Floor, Health Sciences Building, Foresterhill, Aberdeen AB25 2ZD, UK; 2 School of Psychological Sciences and Health, University of Strathclyde, Glasgow, UK

**Keywords:** Osteoarthritis, SF-36, Psychometrics, Item bias, Differential item functioning, Measurement equivalence

## Abstract

**Background:**

The SF-36 is a very commonly used generic measure of health outcome in osteoarthritis (OA). An important, but frequently overlooked, aspect of validating health outcome measures is to establish if items work in the same way across subgroup of a population. That is, if respondents have the same ‘true’ level of outcome, does the item give the same score in different subgroups or is it biased towards one subgroup or another. Differential item functioning (DIF) can identify items that may be biased for one group or another and has been applied to measuring patient reported outcomes. Items may show DIF for different conditions and between cultures, however the SF-36 has not been specifically examined in an osteoarthritis population nor in a UK population. Hence, the aim of the study was to apply the DIF method to the SF-36 for a UK OA population.

**Methods:**

The sample comprised a community sample of 763 people with OA who participated in the Somerset and Avon Survey of Health. The SF-36 was explored for DIF with respect to demographic, social, clinical and psychological factors. Well developed ordinal regression models were used to identify DIF items.

**Results:**

DIF items were found by age (6 items), employment status (6 items), social class (2 items), mood (2 items), hip v knee (2 items), social deprivation (1 item) and body mass index (1 item). Although the impact of the DIF items rarely had a significant effect on the conclusions of group comparisons, in most cases there was a significant change in effect size.

**Conclusions:**

Overall, the SF-36 performed well with only a small number of DIF items identified, a reassuring finding in view of the frequent use of the SF-36 in OA. Nevertheless, where DIF items were identified it would be advisable to analyse data taking account of DIF items, especially when age effects are the focus of interest.

## Background

Osteoarthritis (OA) is one of the most common causes of disability and with aging populations ever more treatments and procedures are being carried out. In order to evaluate the effectiveness of such treatments and procedures, it is essential to have accurate measures of outcome. Without good measures we cannot identify those that do benefit from treatments and those who do not benefit and for whom possibly other less invasive treatments may be more appropriate.

The 36 item SF-36 is the most commonly used generic measure of outcome used in OA [1]. The SF-36 is based on a multidimensional model of health and reflects eight important health concepts. These concepts are limitations in Physical Functioning, Role Limitations due to physical problems, Social Functioning, Bodily Pain, General Mental Health, Role Limitations due to emotional problems, Vitality and General Health Perceptions. There is also a single question on reported Health Transition. While considerable effort has been invested in developing the SF-36 to high psychometric standards, improving the quality of the measure and its interpretation for specific populations, such as OA, is an ongoing scientific task.

An important, but frequently overlooked aspect of establishing the validity of a measure, is to establish if items and measures work in the same way across subgroups of a population? e.g. certain socio-economic groups, or gender. That is, if respondents have the same underlying level of an attribute, such as disability, does the measure give the same score in different populations? or is it biased in some groups. For example, it has been shown that for the Centre of Epidemiology Scale of Depression (CES-D), women are more likely than men to endorse an item about having crying spells even though they have the same underlying level of depression [2]. Thus, while this item may truly reflect differences between men and women in likelihood of crying, it exhibits gender bias with respect to the measurement of depression and scores for women might be inflated compared to men. Hence, apparent group differences in depression scores may be due to measurement bias rather than true differences. Alternatively, where no group differences are found, if DIF items exist then they might mask true group differences. Although a measure may appear equivalent at the measure level, biases may still be present at the individual item level [3]. Thus, item level analyses are now seen as central to establishing measurement equivalence across subgroups of a population [4].

The techniques are known as differential item functioning (DIF) methods and biased items are said to exhibit DIF. DIF items have been identified in health outcome measures with respect to gender, age, race, ethnicity, socio-economic status, language, nationality and health care setting [5]. For example, several items from cognitive screening measures were shown to be poor items for those with low education levels [6]. Use of these items may exaggerate the problems of more deprived individuals.

If DIF items are found in measures in development then these items could be re-written or the item could be removed and an alternative DIF-free item with similar item properties could be substituted. If DIF items are found in an existing measure then it may be preferable to select an alternative measure (with no DIF). If data has already been collected or in situations where there is an established use of a measure such as the use of the SF-36 in OA, then DIF items could be removed and analyses repeated without the DIF items or using an analysis method that can take into account the DIF items; these analyses allow more accurate interpretation of results obtained.

Importantly, it has been shown that SF-36 items may work in different ways for different clinical conditions [7] including some evidence of DIF even between arthritic conditions, (between psoriatic arthritis and rheumatoid arthritis) [8]. Hence, given its frequency of use in OA there is a need to examine items for DIF specifically in an OA population.

Furthermore, it has been shown that cultural differences may impact the validity of SF-36 items [9]. However, DIF in the SF-36 has only been explored in US, Danish, Dutch, Israeli and Chinese patients. It is clearly important to achieve this level of validation for the SF-36 in a UK population. Additionally, only demographic factors have been previously explored; DIF items have been identified for the SF-36 with respect to age, education, gender, race, condition and language in other conditions [7,10-12]. Hence, the aim of the study was to examine DIF in the SF-36 for an OA population with respect to demographic, social, clinical and psychological factors.

## Methods

### Design

Statistical techniques were applied to SF-36 data from a community-based population of UK people, with OA to explore DIF items in the SF-36 with respect to demographic, social, psychological and clinical factors.

### Participants and data collection

The sample comprised a community sample of 763 people who had been diagnosed with OA from 1359 people with hip and/or knee symptoms who completed the SF-36 during a follow-up assessment of health outcome measures as part of the Somerset and Avon Survey of Health Survey (SASH, [13,14]). SASH is a large scale survey of the population aged 35+. The age–sex stratified survey of 28,080 people registered with 40 general practices in Avon and Somerset yielded 2703 people reporting hip and/or knee symptoms at baseline (1994–1995). At follow-up assessment (2002–2003), 763 had OA. diagnosed by a clinician assessing X-rays using the Kellgren-Lawrence classification [15] Written informed consent was obtained from all participants. Ethics approval was obtained from the South West Research Ethics Committee (MREC/01/6/51) and the study was conducted in accordance with the Helsinki Declaration.

### Measures

#### SF36

The 36 item SF-36 is the most commonly used generic measure of outcome used in OA [1]. The SF-36 was developed from the Medical Outcomes study (MOS) based on a multidimensional model of health [16]. The SF-36 is a shorter, 36 item measure that reflects the eight most important health concepts of the MOS. The concepts were limitations in Physical Functioning (10 items), Role Limitations due to physical problems (4 items), Social Functioning (2 items), Bodily Pain (2 items), General Mental Health (5 items), Role Limitations due to emotional problems (3 items), Vitality (4 items) and General Health Perceptions (5 items). There is also a single question on reported Health Transition. Only subscale scores are calculated (i.e. no total score). We used the UK SF-36 version in this study.

#### Validation measures

The Western Ontario and McMaster Universities Osteoarthritis Index (WOMAC [17]) was used to validate the DIF-free SF-36. The WOMAC is the most commonly used disease-specific measure of outcome used in OA. The WOMAC was based on the objective of defining the dimensionality of pain and disability in OA of the hip and/or knee.

#### Grouping factors

Median splits were used where appropriate. The socio-demographic factors explored were gender, age (median = 70.88), social deprivation (measured by Townsend Index group) (median = -1.47), social class (Registrar General classes 1,2,3i v 3ii,4,5) [18] and employment status (paid work v not paid work). The psychological variable investigated was mood assessed using a single item on the EuroQol [19] (no anxiety/depression v moderate or extreme). The clinical factors were Body Mass Index (BMI, underweight/normal/overweight v obese i.e., <30, 30+), the number of affected OA joints (1or 2 v 3 or 4) and type of OA (hip v knee).

### Statistical analysis

First t-tests were used to examine for differences in SF-36 scores between grouping factors. Then the assumption of unidimensionality was tested before testing for DIF.

### Testing assumptions: unidimensionality

Ordinal factor analysis was carried out to explore unidimensionality. We used the FACTOR computer program [20] using common factor analysis with polychoric correlations. Unidimensionality was supported if there were large difference in eigenvalues between factor 1 to 2 and small difference in eigenvalues between 2 and 3 [21]. A widely cited criteria for acceptable unidimensionality is if > =20% variance is explained in first factor [22]. Another commonly reported method for exploring acceptable unidimensionality is by looking at the ratio of the first to second eigenvalue. The first eigenvalue should be significantly higher than the second eigenvalue. If this is 3:1 or 4:1 then there would appear to be a dominant first factor [23]. If both of these criteria were reached unidimensionality was accepted.

The number of factors was also evaluated using the MAP procedure proposed by Velicer (1976) which examines the matrix of partial correlations [24].

#### DIF testing

Details of the method used have been described elsewhere [25]. Briefly, ordinal Logistic Regression (OLR) was used to explore DIF. In DIF analyses it is crucial to control for the underlying attribute that the item is supposed to be measuring since different groups may have different ability levels (i.e. it is necessary to ‘match’ on ability levels). The total score on the relevant subscale was used as the matching variable. Hence, each item was tested within its own subscale.

DIF analysis was carried out for each SF-36 item by testing the effect of the grouping factors and the interaction term (matching variable by grouping factor) once the matching variable has already been added into the model [26,27]. A macro was written in SPSS to facilitate the DIF analysis. Specifically, the following steps for OLR was carried out for each item.

##### Ordinal logistic regression model

i) General procedure for DIF testing

Three OLR models were calculated for each item (the dependent variable).

1) Model 1: The total score (matching variable) was entered as a predictor variable.

2) Model 2: The grouping factor (i.e. binary variable) was added into Model 1 as a second predictor variable

3) Model 3: The interaction (i.e. grouping factor by total) was added into Model 2 as the third predictor variable.

The difference in Chi-square between the Model 1 and Model 3 was tested for the significance as a Chi-Square test with 2 degrees of freedom [26]. If significant, this indicated DIF. The difference in Chi-square between Model 2 and Model 1 gave a test of uniform DIF (same DIF effect over the construct) and the difference in Chi- square between Model 3 and Model 2 gave a test of non-uniform DIF (uneven DIF effect over the construct) [26].

##### Significance testing and item level effect sizes

Different criteria have been suggested to classify items as exhibiting DIF and as previously described [25] we classified DIF items using the criteria proposed by Swaminathan and Rogers (SR, 1990) [27]. Swaminathan and Rogers use a criteria of p < 0.05 for the difference in Chi-square between Model 3 and Model 1. Bonferroni corrections were also applied to minimise Type 1 error [10,28]. For uniform DIF the odds ratios were calculated to examine the direction of the bias.

ii) Assumption testing: Proportional Odds

An assumption of ordinal logistic regression is that the parameter coefficients are equivalent across the levels of the dependent variable (i.e. proportional odds). If for any model the assumption of proportional odds was violated then k-1 dichotomous variables were created for that item where k is the number of response categories.

##### Purification

If DIF items were found then they were removed from the total score (i.e. the matching variable) and all the analyses for the items in that measure re-run. As standard, the item with DIF was included in the total score used in testing that item as this has been shown to reduce bias [29]. Purification was an iterative process so the analyses may be re-run a number of times until no changes in identified DIF items were seen on two consecutive analyses.

##### Effects of covariates

Where DIF items were identified, the analyses were repeated (steps a-b above) with age additionally entered as a covariate in the logistic regressions to explore if apparent DIF effects in other grouping factors were confounded by age.

##### Examination of the impact of DIF at the measure and subscale level

Modified measures were constructed with DIF items removed and compared to the original measure or subscale. The effect of DIF on group differences was explored using t-tests to see if different conclusions would result if a DIF-free measure was used. Also the difference in significance between the tests was explored by repeated measures ANOVA and exploring the interaction between the two different total scores and the grouping factor i.e. does the effect size change by a significant amount depending on the total used. All totals were recalculated as averages due to the different number of items in each total.

##### The validity and reliability of DIF-free measure

The validity and reliability of DIF-free measure was explored by carrying out standard psychometric tests. Construct validity was explored by examining the relationship of the DIF free measures with other subscales from the WOMAC. Cronbach’s alpha was calculated for the original measure and for the DIF free measure.

##### Power

Based on Crane’s (2006) [28] suggestion for number of participants in each subgroup, we required at least 80 participants per subgroup (based on the maximum of 6 response categories). All subgroups had more participants than the minimum required.

## Results

### Demographics

The participant’s characteristics are presented in Table 1.

**Table 1:** Participant characteristic table

Gender (male, %, (n))	43.3% (330)
Age (Mean years, s.d.)	69.5 (10.0)
Marital status (married, %, (n))	72% (543)
Ethnicity (white, &, (n))	99.1% (750)
Paid employment (yes, %, (n))	33.7% (247)
Social class (%, (n))	
I	5.7% (43)
II	33.8% (256)
IIINM	19.0% (144)
III M	23.4% (177)
IV	14.7% (111)
V	3.4% (26)
Townsend quintiles (lower = most affluent)	
20%	-2.7
40%	-1.9
60%	-0.80
80%	1.4
Mood (mod or severe/none; %, (n))	30%/70% (218/534)
BMI (mean(s.d.))	29.2 (5.3)
Affected joints (n)	
Hip OA	487
Knee OA	612
Both hip and Knee OA	336
Hip OA only	151
Knee OA only	276
No of affected hip/knee joints (1/2v3/4;%, (n))	57%/43% (218/435)
SF-36 subscales^	
Physical functioning (mean(s.d.)	50.0% (29.0)
Pain (mean(s.d.)	42.3% (20.0)
Role physical (mean(s.d.)	39.2% (41.7)
Social (mean(s.d.)	71.8% (28.3)
Role emotional (mean(s.d.)	57.7% (42.9)
Mental health (mean(s.d.)	73.9% (18.1)
Vitality (mean(s.d.)	50.8% (21.3)
General health (mean(s.d.)	59.6% (21.2)

^ higher = better function.

Being older was associated with being female (t(706) = -2.89 p = 0.004), being in lower social deprivation group (Townsend scores)(t(208.7) = -2.12 p = 0.03), having better mood (t(327.9) = 1.73 p = 0.08), more affected joints (t(543) = -4.43 p < 0.0005), knee only OA (compared to hip only OA) (t(208.7) = -2.12 p = 0.03) and not working (t(706) = -20.0 p < 0.0005).

### Testing assumptions: unidimensionality

The ordinal factor analysis supported the unidimensionality for all subscales of the SF-36 with large difference in eigenvalues between factor 1 to 2 and small difference in eigenvalues between 2 and 3. Only one dimension was also suggested from the MAP procedure for all of the subscales. Hence there was evidence of unidimensionality for all subscales (see Table 2).

**Table 2:** Ordinal FA

**SF-36 subscale**	**Factor1 eigenvalue**	**% variance**	**Factor2 eigenvalue**	**% variance**	**Factor3 eigenvalue**	**% variance**	**MAP: number of dimensions**	**N**
Physical	7.83	78.3	0.50	5.0	0.47	4.7	1	734
Role phys	3.65	91.3	0.15	3.8	0.13	3.3	1	749
Role emot	2.71	90.3	0.19	6.3	0.10	3.3	1	742
Mental	3.36	67.1	0.67	13.3	0.46	9.2	1	753
Vitality	2.94	73.4	0.61	15.2	24.1	6.0	1	754
Gen health	3.27	65.5	0.65	13.0	0.56	11.3	1	756

#### DIF items

DIF items were found across 8 of the 9 grouping factors (the exception being the grouping factor ‘number of affected joints’). Of the 35 items, 16 items showed DIF for at least one of the grouping factors (without counting the mental subscale items for the mood grouping factor as these should exhibit DIF) (see Table 3).

**Table 3:** DIF analysis: CHI SQUARE values for overall DIF (model 3-model1) with uniform and non-uniform DIF identified

**Item**	**Age**	**Gender**	**Social class**	**Social deprivation**	**Employ**	**Mood**	**BMI**	**No. joints**	**Hip/Knee**
*Physical functioning*									
PF1 vigorous activities	**19.21**^**NU**^	2.26	1.26	5.24	3.99	0.23	**13.80 **^**NU**^	0.99	0.93
PF2 moderate activities	1.98	1.32	5.90	5.15	1.47	3.93	0.21	4.77	1.50
PF3 lift/carry groceries	0.53	**29.08 **^U^_*_	1.13	0.39	2.40	1.90	1.03	2.53	2.98
PF4 several flight stairs	1.96	10.17	4.24	5.17	5.14	4.94	2.13	3.00	5.63
PF5 one flight stairs	2.87	0.49	2.33	1.91	0.79	0.54	3.31	1.44	1.67
PF6 bend/knee/stoop	**16.73 **^**NU**^	5.58	4.74	2.31	4.42	1.21	5.43	1.024	0.21
PF7 walk more mile	4.04	2.63	2.85	0.92	2.75	2.09	0.85	1.39	0.65
PF8 walk half mile	0.37	0.41	3.37	3.01	1.02	1.48	0.50	0.57	7.25
PF9 walk 100 yards	10.73	0.20	2.87	6.53	0.00	3.01	2.40	4.07	0.81
PF10 bathing or dressing	1.01	4.36	2.04	3.71	11.01	**12.67**	6.26	1.80	1.49
*Pain*									
P1 intensity of bodily pain	8.55	0.24	8.21	1.60	**32.45 **^NU^_*_	4.65	4.10	0.73	4.59
P2 extent pain interfere normal work	5.78	7.66	**10.34**^U/NU^_*_	5.0	**10.27**	5.92	2.05	0.99	7.18
*Role physical*									
RP1 cut down time on work/act.	1.74	0.63	4.20	**10.26 **^NU^_*_	**17.13**^NU^_*_	0.44	0.05	2.65	**9.18 **^**NU**^
RP2 accomplished less	**10.98 **^**U**^	1.05	1.43	0.89	0.72	0.48	0.44	2.55	4.14
RP3 limited in kind of work/activities	1.58	2.08	1.75	3.62	0.72	8.57	0.28	6.54	0.16
RP4 difficulty performing work/act.	2.10	1.69	2.80	1.04	**10.52 **^**U**^	5.42	0.15	1.26	0.80
*Social functioning*									
SF1 interferes with social activities	**14.95 **^**NU**^	3.82	9.05	0.87	**19.50 **^NU^_*_	0.43	1.71	0.74	1.46
SF2 time interferes with social act.	9.67	9.57	1.33	5.95	7.16	4.80	1.83	0.22	4.78
*Role emotional*									
RE1 cut down time work/activities	0.42	0.25	5.75	1.40	1.14	0.12	1.03	0.32	0.97
RE2 accomplished less	6.68	0.35	0.18	2.00	6.45	4.06	3.46	5.45	9.32 _*_
Not as careful work/activities	6.94	1.15	1.66	2.30	2.66	5.00	4.85	4.21	0.97
*Mental*									
MH1 nervous	3.05	**14.50 **^U^_*_	4.19	0.74	5.40	**18.11 **^NU^_*_	1.53	4.24	2.31
MH2 down in dumps	4.14	0.98	**12.31**^U^_*_	0.65	0.95	7.28	6.96	2.32	1.24
MH3 calm and peaceful	8.48	6.54	1.23	4.00	6.36	**46.33 **^NU^_*_	11.48	4.63	9.75
MH4 downhearted and low	2.39	1.11	4.13	10.24	5.09	**14.35 **^NU^_*_	8.22	0.02	1.29
MH5 happy person	0.93	1.64	8.36	3.18	9.18	**47.19 **^NU^_*_	3.82	0.27	2.60
*Vitality*									
V1 full of life	6.93	0.16	3.25	7.25	4.56	7.48	3.09	4.95	0.28
V2 energy	**10.91 **^**U**^	0.36	0.17	1.10	1.67	5.35	2.57	2.70	0.44
V3 worn out	**8.82 **^**U**^	0.33	0.05	3.25	**10.45 **^**U**^	0.34	1.68	4.38	0.45
V4 tired	5.49	8.19	2.48	2.20	7.07	2.59	0.20	0.91	0.88
*General health*									
GH1 health in general	9.12	2.30	6.04	2.21	8.77	3.05	1.49	2.48	2.66
GH2 get ill more easily than others	2.26	3.70	1.01	6.64	1.39	**12.03 **^U^_*_	1.62	3.71	3.16
GH3 as healthy as anybody I know	2.47	5.74	6.31	2.51	5.02	3.97	2.41	2.10	0.26
GH4 except health to get worse	4.36	10.26	6.61	10.71	4.47	3.46	1.68	4.12	2.41
GH5 my health is excellent	0.94	1.05	0.91	2.49	1.10	9.85	2.77	7.90	0.23

Where the item has been dichotomised, the larger value is entered in the table.

KEY: U = uniform. NU = non-uniform, * = still significant after adjusting for age.

Boldface=significant DIF.

**Figure 1:**
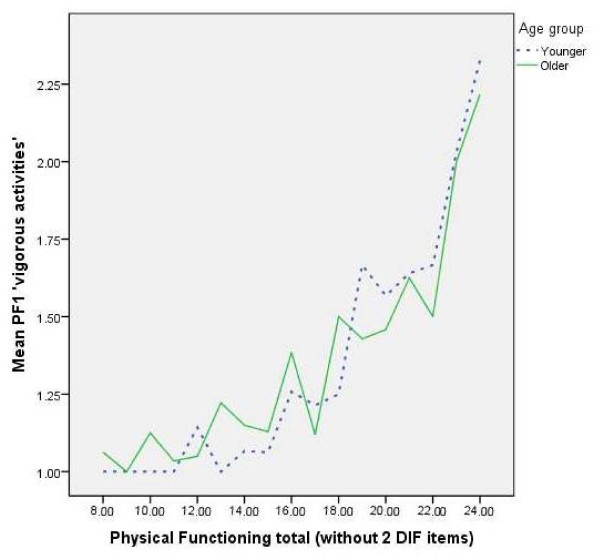
**Line chart of PF1 ‘*vigorous activities’ *** **by physical functioning score by age group.**

The greatest number of DIF items were identified for age and employment status (each with 6 items). For age, items that showed DIF were, from the physical functioning subscale, item PF1 concerning ‘*vigorous activities’* and PF6 ‘*bending kneeling, stooping’*; from the role physical subscale, RP2 ‘*accomplishing less’*, from the social functioning subscale SF2 ‘*interference with social activities’* and from the vitality subscale, V2 ‘*energy*’ and V3 ‘*worn out’*. The items RP2 ‘*accomplishing less’* and V2*‘energy’* had uniform DIF with older people reporting more limitations than they would have with an unbiased item, but the item V3 *‘worn out’* showed uniform DIF in the other direction with older people reporting less limitation than their overall level would suggest. The other items that showed DIF by age had non-uniform DIF. It appeared that older people with good overall physical functioning reported more problems with vigorous activities than younger people with the same level of overall physical functioning. Whereas older people with mid range or poor overall physical functioning reported fewer problems with vigorous activities than younger people with the same level of overall physical functioning (see Figure 1).

**Figure 2:**
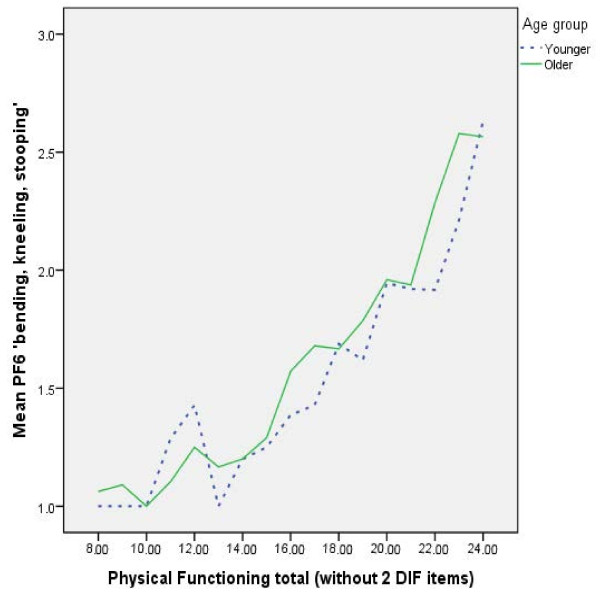
**Line chart of PF3 *‘bending, kneeling, stooping’ *** **by physical functioning score by age group.**

However, for PF3 ‘*bending, kneeling and stooping’* older people with very poor overall physical functioning reported having more problems with bending, kneeling and stooping than younger people who also had poor physical functioning, whereas older people with mid-range to very good physical functioning reported having fewer problems with bending, kneeling and stooping than younger people who also had same level of overall physical functioning (see Figure 2).

Older people with mid-range to very good overall social functioning responded to having fewer problems with SF1 ‘*interference with social activities’* than younger people who also had same level of overall social functioning, whereas older people with poor overall social functioning responded to having more problems than younger people who also had same level of overall social functioning.

For employment status, DIF was also identified for 6 items. The DIF items were both the items in the pain subscale (P1 and P2); RP1 *‘cut down time on work and activities’* and RP4 ‘*difficulty performing work and activities’* from the role physical subscale; and SF1 ‘*interference with social activities*’ from the social functioning subscale and from the vitality subscale V3 ‘*worn out’.* Uniform DIF was identified for employment status with those not working reporting less ‘*difficulty performing work and activities’* and being less *‘worn out’* than those people who were working who also had same level of functioning on the relevant subscales.

The other items that showed DIF by employment status had non-uniform DIF. It appeared that for at the worse end of the pain subscale, those not working reported greater intensity of pain (P1) than those working whereas over the rest of the subscale those not working reported less pain than those working at similar overall pain score.

**Figure 3:**
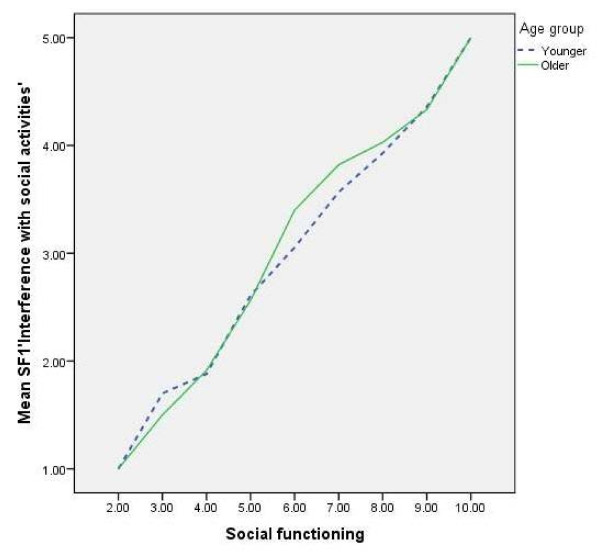
**Line chart of SF1 *‘interference with social activities’ *** **by social functioning score by age group.**

The other pain (P2) item didn’t quite reach significance for uniform DIF (although significant overall DIF) but it appeared that those not working reported fewer problems across the whole range than those working with the same level of overall pain. For the item RP1 *‘cut down time on work and activities’* over most of the construct range there was little difference between the employment groups, however at the better end of the subscale those not working reported fewer limitations than those working and with similar levels of function. For those with similar social functioning in the mid-range of the subscale, those not working reported fewer limitations than those working on the item SF1 ‘*interference with social activities*’ with responses similar over the rest of the subscale (see Figure 3).

After adjusting for age, 3 items still showed DIF for employment status, P1*‘intensity of bodily pain’*; RP1 ‘*cut down time on work and activities’* and SF1 ‘*interference with social activities*’.

For the other grouping factors, 2 items were identified as having DIF for gender PF3 ‘*lifting/carrying groceries’* and M1 *‘nervous’*; 2 items for social class (P2 ‘*extent pain interferes with normal work’* and M2 ‘*down in the dumps’*), 1 item for deprivation (RP1 ‘*cut down time on work and activities’)*, 2 items for mood (excluding mental functioning subscale, PF10 ‘*bathing and dressing’* and GH2 *‘get ill more easily than others’*), 1 item for BMI (PF1 ‘*vigorous activities’)*, and 2 items for Hip v Knee OA (RP1 ‘*cut down time on work and activities’* and from role emotional RE2 *‘accomplished less’*). Only gender had consistently uniform DIF; women were more likely to respond as having more limitations carrying groceries and nervousness than men, at the same the overall level of limitation. Uniform DIF was also identified for M2 ‘*down in the dumps’,* with those in the lower social class group more likely to report more problems than those in the higher social class group at the same level of overall mental health. The other DIF item for social class P2 ‘*extent pain interferes with normal work’* showed non-uniform DIF, with similar responses to the item between the groups over most of the low to mid range, but across the mid-range those in the lower social class group reported more limitations than those in the higher social class group. However, at the high end of the subscale, those in the lower social class group reported less pain interference on this item than those in the higher social class group

Uniform DIF was also identified for GH2 *‘get ill more easily than others’* with those with low mood reporting more difficulties than those without low mood who also had same level of overall general health.

The item PF10 ‘*bathing and dressing’* showed non-uniform DIF for mood; those with low mood and at the best end of physical functioning reported fewer problems than those without low mood with the same level of overall physical functioning. However over the rest of the scale those with low mood reported more difficulties than those without low mood at similar levels of physical functioning.

For hip v knee OA, those with hip OA reported better scores on the item RP1 ‘*cut down time on work and activities ’*than those with knee OA with the same scores on the role physical subscale across the whole range of role physical; differences with knee OA were even greater at the better end of the subscale. For the item RE2 ‘*accomplished less’* from the role emotional subscale, those with hip OA reported more limitations across the whole range of the subscale than those with knee OA with the same scores on the role emotional subscale; differences with knee OA were even greater at the more limited end of the subscale.

After adjusting for age, there were no changes in the identified DIF items for gender, social class and deprivation, however only 1 DIF item remained for mood, GH2 ‘*get ill more easily than others’* and one item for Hip v Knee, RE2 ‘*accomplished less’* and the one item for BMI, PF1, was no longer being identified with DIF (see Table 3).

### Impact

#### Testing for group differences using original and DIF-free measures

If DIF-free measures were used, then the apparent conclusions for two group differences would have changed. Using the original scoring method, older people had worse scores on physical functioning, role physical, role emotional but better scores on mental functioning and general health than the younger group. When the DIF-free totals were used older people had significantly worse scores for social functioning compared to the younger group. Although the conclusions did not change for the other DIF-free subscales by age-group they all had significant changes in effect size.

Those in the lower social group, with worse deprivation scores and not working had worse scores on all subscales (other than for mental functioning in the employment group). However when the role physical subscale was DIF adjusted, there was no longer a significance between deprivation groups with a trend of a significant change in effect size. No other DIF adjustments changed conclusions although most changed the effect size.

With the original scoring, women had worse physical functioning, pain, social functioning, mental functioning and vitality. When the DIF-free totals no conclusions changed but the effect sizes significantly changed.

High BMI was associated with worse scores on all subscales but no conclusions changed with DIF adjustment although there was a significant change in effect size.

Those with lower mood had worse scores on all of the subscales but there was no change in conclusion where DIF free totals were used and no change in effect size.

Mean subscale scores were not significant different for those with one or two affected joints compared to those with 3 or 4 affected joints nor for those with hip OA compared to knee OA. These conclusions did not change where DIF adjusted scores were used and no change in effect size was found (see Table 4).

**Table 4:** Comparison of original and DIF-free SF-36 subscales by grouping factors (t-value and effect size)

**SF-36 sub-scale**	**Age**	**Gender**	**Social class**	**Social dep**	**Employment status**	**Mood**	**BMI**	**No joints**	**Hip/Knee**
**Phys**									
Original	4.80***	3.71***	3.82***	3.50***	8.53***	7.63***	7.17***	1.39 ns	0.09 ns
No DIF	4.89***	3.24***				7.30***	7.14***		
Effect size	p = 0.015	p < 0.0005				p = 0.21 ns	p = 0.02		
**Pain**									
Original	-1.06 ns	1.93*	5.63***	2.99***	3.77***	6.18***	6.67***	-0.23 ns	1.35 ns
No DIF			5.95***		^2.72***/4.43***				
Effect size			p = 0.09		p = 0.006/0.006				
**Role Phys**									
Original	5.40***	1.70 ns	3.27***	2.27*	8.89***	5.13***	4.22***	1.29 ns	0.34 ns
No DIF	4.82***			**1.81 ns**	9.24***				0.16 ns
Effect	p = 0.02			***p = 0.07***	p = 0.002				p = 0.42
**Social**									
Original	**1.54 ns**	2.61***	4.13***	4.09***	5.37***	12.68***	4.69***	0.23 ns	-0.45 ns
No DIF	2.17*		4.01***		6.20***				
Effect	**p = 0.04**		p = 0.37		p = 0.002				
**Role Emot**									
Original	2.92***	1.66 ns	3.27***	3.57***	4.81***	8.98***	3.16***	-0.12 ns	0.75 ns
No DIF									1.22 ns
Effect									p = 0.12
**Mental**									
Original	-2.55*	2.89***	3.45***	3.49**	0.04 ns	22.24***	2.94***	-1.13 ns	0.12 ns
No DIF		2.12*	2.88***						
Effect		p = 0.003	p < 0.0005						
**Vitality**									
Original	0.12 ns	2.55*	2.31*	3.15***	2.95***	12.29***	4.96***	0.26 ns	-0.55 ns
No DIF	0.14 ns				3.53***				
Effect	p = 0.22				p = 0.001				
**Gen health**									
Original	-2.56*	-0.80 ns	4.42***	4.34***	2.42*	11.14***	3.96***	-1.59 ns	-1.25 ns
No DIF						10.17***			
Effect						p = 0.80			

***p < 0.01, **p < =0.01, *p < =0.05. ^ as both pain items exhibit DIF, each is taken out and t-test run. Social Dep: social deprivation (Townsend) score.

Boldface=different conclusion reached if measure DIF-free.

### The validity and reliability of DIF-free measures

The removal of the DIF items from the subscales (of more than two items) only resulted in small changes to Cronbach’s alpha except for the vitality subscale where alpha reduced from 0.86 to 0.70. This was probably due to the number of items halving from four to two items. The strength of correlations of the SF-36 physical functioning and pain subscales with the physical and pain WOMAC subscales were only slightly reduced (not shown).

## Discussion

Overall, the SF-36 preformed well. However, each subscale showed some evidence of DIF by at least one grouping factor: physical function (4/10 items), Pain (2/2), Role-Physical (3/4), Social functioning (1/2), Role emotional (1/3), Mental health (excluding mood as a grouping factor, 2/5), Vitality (2/4) and General health (1/5). Previous studies that explored DIF in the SF-36 by socio-demographic factors also found many subscales with DIF items. DIF items were found in all the SF-36 subscales of a US general population and in all except social functioning and role emotional in a chronic condition population [10]. Other US based studies have examined particular sub-scales for the presence of DIF; DIF items were found in the physical functioning and mental health subscales in people with chronic diseases [30] and in the physical functioning subscale in people with fibromyalgia [12]. DIF items were also found when only the general health subscale was examined in a Danish general population study [11].

DIF items were found across all of the grouping factors except for ‘number of affected joints’. Of the 35 items, 16 items showed DIF for at least one of the grouping factors. DIF was most commonly found for age and employment status (6 items each) and so DIF may be less of a problem within samples that are homogeneous for employment or age although when controlling for age, there was less evidence of DIF for employment (3 items). In previous studies, more items showed DIF for age and education than for other grouping factors [10], although items with DIF were also identified for gender, marital status and income [30].

In common with previous US general and chronic condition populations studies, we identified DIF by age for items ‘*Vigorous activities*’ and ‘*Bending, kneeling stooping’* in the physical functioning subscale [10,30] and for the items in the vitality subscale *‘energy’* and ‘*worn out’*[10]. We also identified the item ‘*Lifting, carrying groceries’* from the physical functioning subscale for DIF by gender in common with the study of people with chronic diseases [30]. We also found six items that exhibited DIF by employment but this was not found in the only other study that included employment status [30]. These six items included both the items from the pain subscale. The other main difference between the current study and previous studies was for the general health subscale where previously most of the items showed DIF for age [10,11], education, gender and race [10], while in our UK OA population no DIF by socio-demographic factors was identified. We also found a small number of DIF items across the subscales that were not identified in other studies. There are many possible explanations for why we have found different DIF items to the other studies, this may be as none of the previous studies focused solely on an OA population and hence differences may be attributed to the particular difficulties and challenges faced by people with OA. Additionally, this is the first study to examine the SF-36 for DIF in a UK population. Also, many of the previous studies used general population datasets which have wide age ranges, whereas, the present study employed an older population only.

At test-level, in common with other studies, few changes in conclusions were found when DIF free subscales were compared to the original subscale although, in most cases, effect sizes significantly changed. Two changes of conclusion were made after re-analysis; one for comparisons by age and the other for a comparison by social deprivation (Townsend index) score. Hence, we would suggest for these comparisons, it would be particularly advisable to re-run analyses without the DIF items or use an analysis method that takes account of the DIF items. As many changes in effect size were found between the original and DIF free scales, it would also be prudent to re-analyse for all the comparisons where DIF items were found.

These results have implications for interpretation for SF-36 results and for the understanding of the process and pattern of disablement in OA. Some of the findings suggest that individual aspects of OA may affect different groups in different ways. So for example, women are more likely to report being nervous and having difficulty in lifting and carrying groceries than their actual level of function would suggest. There also appear to be differential effects on older people; they are more likely to report accomplishing less and having low energy than younger people with similar limitations of function, but are less likely to describe themselves as worn out. If older people have overall poor function, then they reported having fewer problems with vigorous activity than their level of overall physical functioning would suggest but report having more problems with bending and kneeling and with interference in social activities than their level of overall function would suggest. This pattern of findings is compatible with models of ageing that suggest older people select activities they wish to preserve and work to optimise their performance of the selected activities [31], but have more problems with activities that are essential rather than chosen.

Employed people, reported more work-related difficulties and in feeling more worn out than would be expected for their level of functioning on the respective subscales compared with those who are not in employment. In addition, other limitations may be more pronounced for employed people with poor function. Again this suggest that the methods of accommodating to impairments may be important in determining the pattern of limitations and that continuing in employment may bring a specific pattern of difficulties which follow logically from the work itself.

In the study we took the approach of removing the DIF items. However removing items may affect content validity of the measure and comparability with other studies. Using more complex Item Response Theory-based analyses, DIF items do not need to be removed as adjusted scores can be calculated for each subgroup. Alternatively, researchers may choose to stratify by gender, age etc. in the design or analysis of studies using the SF36. If the measure is in development, an alternative to deleting the DIF items, may be to substitute similar but DIF-free items either by re-writing, or choosing an alternative item with similar item properties. Re-writing could be facilitated by the identification of the source of DIF, for example by cognitive interviewing or by reviewing the item by groups of experts.

The study has limitations. The sample was a community sample and thus had relatively mild OA compared to, say, an arthroplasty sample, hence the generalisability of these results to all levels of OA would need exploring. We created some groups by using median splits and it is possible that other splits may have produced different results. Additionally although we adjusted for age, it is possible that DIF effects could be due to differences in other covariates between groups and it is possible that there are not real differences in the underlying response probabilities

We also carried out a large number of statistical tests and although we applied a Bonferroni correction it is possible that some findings were due to chance and thus replication would be desirable. Also 2 scales (pain and social functioning) only had 2 items, so could not be purified and the total score was based on a very small number of items.

In this study we used OLR to explore DIF due to the accessibility, flexibility and practicality of this method. However, another approach to DIF detection is to use the more complex item response theory (IRT) approach including Rasch models. There is still much debate over the advantages and disadvantages over different methodological approach to DIF [31-33]. IRT does have advantages, in particular the use of the latent variable as the matching variable rather the use of sum scores in OLR. However, IRT is a complex statistical method requiring the use of specialist software and yet produces similar results to OLR. Additionally, IRT requires good model fit as poor model fit can contribute to false DIF detection and yet the methods for assessing model fit are not fully established [32,33]. However, it is possible that we may have obtained different results if an alternative DIF method was used. It is also possible that by using different significance criteria for the OLR method we may have reached different conclusions.

## Conclusions

Overall a small number of DIF items were identified, a reassuring finding in view of the frequent use of the SF-36 in OA. Although individual items exhibited DIF, this rarely extended to the measure level, although in most cases the effect sizes changed significantly. Nevertheless, where DIF items were identified it would be advisable to analyse data taking into account DIF items especially when age effects are the focus of interest. The results demonstrate the importance of DIF detection as a standard part of validity testing for measures of health outcome.

## Competing interests

The authors declare that they have no competing interests.

## Authors’ contributions

BP participated in the conception and design of the study, the analysis and the drafting and revision of the manuscript. MJ participated in the conception and design of the study and the drafting and revision of the manuscript. DD contributed to the interpretation of the data and revision of the manuscript. All authors read and approved the final manuscript.

## Pre-publication history

The pre-publication history for this paper can be accessed here:

http://www.biomedcentral.com/1471-2474/14/346/prepub
